# A count-based decision method for target blood pressure achievement in home blood pressure monitoring data interpretation for clinical practices

**DOI:** 10.1038/s41598-022-04913-9

**Published:** 2022-03-10

**Authors:** Jinho Shin, Yonggu Lee, Sang-Hyun Ihm, Jeong-Hun Shin, Hyun-Jin Kim, Byung-Sik Kim, Hwan-Cheol Park, Young-Hyo Lim, Jin-Kyu Park, Ran Heo, Woo-Hyun Kim

**Affiliations:** 1 Division of Cardiology, Department of Internal Medicine, Hanyang University Medical Center, Wangsiprio 222, Seongdonggu, Seoul, Republic of Korea; 2 Division of Cardiology, Department of Internal Medicine, Hanyang University Guri Hospital, Gyeong-choonro 153, Guri City, Gyeonggido Republic of Korea; 3 Division of Cardiology, Department of Internal Medicine, The Catholic University of Korea, and Catholic Research Institute for Intractable Cardiovascular Disease, College of Medicine, The Catholic University of Korea, Seoul, Republic of Korea

**Keywords:** Cardiology, Medical research, Hypertension

## Abstract

Home blood pressure (HBP) is useful to decide whether blood pressure (BP) is controlled. However, applying HBP to daily clinical practices is still challenging without easy access to the average HBP. Therefore, we developed a simple method to make a quick decision regarding the controlledness of HBP through high BP counts. We simulated 100 cases of HBP series for each combination of 3 numbers of BP readings (*K* = 16, 20, 24) and 4 levels of the standard deviations (SDs = 5, 10, 15, 20). A high BP was defined as an individual BP ≥ 135/85 mmHg, and an uncontrolled HBP was defined as a mean HBP ≥ 135/85 mmHg. Validation for the decision method was conducted using actual HBP data. The C-statistics and the accuracy of the high BP counts for the uncontrolled HBP were generally high (> 0.85) for all combinations of *K*s and SDs and decreased as SDs increased but remained steady as *K*s increased. In validation, the C-statistic of the high BP count-to-total BP reading (C/T) ratio was 0.985, and the C/T ratio ≥ 0.5 showed a sensitivity of 0.957, a specificity of 0.907, and an accuracy of 0.927. The count-based decision method can provide an accurate quick assessment of the controlledness of HBP.

## Introduction

Home blood pressure (HBP) is useful to detect hypertension and to provide guidance on the treatment of hypertension^1^. HBP monitoring (HBPM) can be used to identify masked hypertension and white-coat (uncontrolled) hypertension^2–4^. HBPM is also effective in improving patient adherence, allowing patients to engage in self-management or self-monitoring of blood pressure (BP)^5^. HBP correlates better with target organ diseases and cardiovascular outcomes than clinic BP^6,7^. Therefore, guidelines recommend using HBPM in diagnosing hypertension and guiding antihypertensive therapy^3,8^.

However, employing HBPM in daily clinical practice may impose a substantial burden on physicians because the calculation of mean BP from HBP records could be cumbersome and time-consuming, especially when assists or resources are insufficient in the clinic. Physicians could not make a quick decision regarding whether a patient achieved target BP without a ready-to-use analysis result of HBP records. This difficulty acts as a barrier to stop broadening the use of HBPM in daily practice^9,10^. Some HBPM devices provide built-in systems allowing the storage and transfer of BP records through Bluetooth or wireless internet technology. However, roughly half of people worldwide still have no access to the internet, and the rates of internet accessibility are much lower in developing and underdeveloped countries^11^, and the rates of daily internet utilization are even lower than the rate of internet access. Furthermore, these new HBPM devices with internet or mobile connectivity may not be familiar to elderly individuals who comprise the absolute majority of hypertensive patients, and the majority of HBPM devices still do not provide such functions.

In this regard, despite the improvement in HBPM device connectivity, physicians still need simple and accurate methods to quickly interpret HBP records to broaden the use of HBPM in daily clinical practice. Therefore, we created a simple count-based method to make a quick decision regarding whether BP was controlled using simulated HBPs and validated the count-based method in a real-world HBPM dataset.

## Methods

### Simulation

Simulated HBPs were generated using statistical software R (R Core Team, R Foundation for Statistical Computing, Vienna, Austria) and RStudio (RStudio Team, Rstudio, BPC, Boston, MA, US). Here, we describe important R codes used to generate the simulated HBPs below the relevant paragraphs in a different font style to improve the understanding and reproducibility of the simulation method. The complete R codes used to perform the entire simulation, data processing, validation, statistical analyses, and result illustrations are provided in Supplementary Data S1.

1. Simulation of mean HBPs.

We produced 100 random sample cases of mean SBP between 130 and 140 mmHg and mean DBP between 80 and 90 mmHg using “runif”, a random number generating function as follows, to generate individual HBP samples around the mean SBPs and DBPs later, because it will be most difficult for physicians to decide whether HBP achieves the target BP level, 135/85 mmHg, when the mean HBP levels were close to the target BP level.

set.seed (1234)

N <− 100

M.sbp <− runif(n = N, min = 130, max = 140)

M.dbp <− runif(n = N, min = 80, max = 90)

2. Simulation of HBP series according to the numbers of HBP readings and SD levels.

Guidelines recommend obtaining HBPM for 5–7 days serially^2,3^, and some researchers recommend excluding BP readings obtained on the first day of a series in mean BP calculation to reduce the reproducibility^12^. Consequently, we assumed that 16–24 HBP readings in a month were required to standardize HBPM. Therefore, we generated 16, 20, and 24 SBP and DBP readings per 1 mean SBP and DBP sample.

For each mean SBP and DBP, a set of *K* SBPs and DBPs was simulated using “rnorm”, a function generating random numbers with a normal distribution at 4 different SD levels, which were 5, 10, 15, and 20 mmHg for SBP and 3.5, 7.0, 10.5, and 14 mmHg for DBP (70% of the SDs for SBP). All sets of simulated HBPs are provided in Supplementary Data S2. Here is an example of R codes for *K*=16, as follows:

N <-100

K <-16

SD <-c(5, 10, 15, 20)

sd_label <-c("SD = 5", "SD = 10", "SD = 15", "SD = 20")

mk<− matrix (NA, ncol = k, nrow = N)

sbp <− list(mk, mk, mk, mk)

dbp <− list(mk, mk, mk, mk)

names(sbp)<-sd_label; names(dbp)<-sd_label

dimnames <-list(paste0("Pt", 1:N), sd_label)

msd <− matrix(NA, ncol = NROW(SD), nrow = N, dimnames = dimnames)

meansbp <− msd; meandbp <-msd

shtn <− msd; dhtn <− msd

nsbp135 <-msd; ndbp85 <-msd

for (j in 1:NROW(SD)){

for (i in 1:N){

sbp [[j]][i,]<-rnorm(nk, mean = M.sbp[i], sdSD[j])

dbp [[j]][i,]<-rnorm(nk, mean = M.dbp[i], sdSD[j]*0.7)

nsbp135[i,j]<-NROW(which(sbp[[j]][i,]>=135))

ndbp85[i,j]<-NROW(which(dbp[[j]][i,]>=85)) }

}

meansbp[,j]<-apply(sbp[[j]], 1, mean)

meandbp[,j]<-apply(dbp[[j]], 1, mean)

shtn[,j]>-ifelse(meansbp[,j]>=135, 1, 0)

dhtn[,j]>-ifelse(meandbp[,j]>=85, 1, 0)

}

3. Definition of a high BP reading, high BP count, and uncontrolled BP.

A high SBP/DBP reading was defined as an individual SBP ≥ 135 mmHg and/or DBP ≥ 85 mmHg, and a high SBP/DBP count was defined as the number of high BP readings in an HBPM series. An uncontrolled SBP/DBP was defined as a mean SBP ≥ 135 mmHg and/or a mean DBP ≥ 85 mmHg. Diagnostic performances of the count-based decision for uncontrolled BP were assessed using receiver operating characteristic (ROC) curves and C-statistics. The best threshold for high BP counts was decided at the maximum value of Youden’s J-indices. In addition to the diagnostic performances at the best threshold, we also investigated the diagnostic performances of high BP counts for uncontrolled BP at intuitive fixed cutoff values. For physicians to use the cutoff values easily, the cutoff values were set to half of the *K* values, 8, 10, and 12.

4. Assessment and comparison of the diagnostic performances for uncontrolled BP.

Sensitivity, specificity, positive predictive value (PPV), negative predictive value (NPV), correct classification rate (CCR), and C-statistic were assessed as diagnostic performance indices of high BP counts. Although Delong’s method can be employed to generate the confidence intervals (CIs) for C-statistics and to compare C-statistics between 2 groups using their CIs, there has been no established method to compare C-statistics and other diagnostic performance indices among 3 or more groups. Therefore, we employed a bootstrap resampling method to create the distributions of the diagnostic performance indices. For each combination of the 3 *K*s (16, 20, and 24) and 4 SD levels (5, 10, 15, and 20), we created 2,000 bootstrap samples of the diagnostic performance indices using the “boot” function. Subsequently, 24,000 bootstrap variations of the diagnostic performance indices for SBP were created for a total of 12 combinations of *K*s and SDs and another 24,000 bootstrap variations for DBP as follows:

*#For K16*

roc.sbp.k16 <-list(NA, NA, NA, NA)

bootraw.auc.sbp.k16 <-list(NA, NA, NA, NA)

boot.auc.sbp.k16 <-matrix(NA, nrow = 2000, ncol = 4)

*#For ROC curve objects*

for (i in 1:4) {

 roc.sbp.k16[[i]]<-roc(shtn[,i]~nsbp135[,i], ci=T, auc=T)

}

*#For Bootstrap resampling*

fx<-function(data, indices, x, y){

d<-data[indices, ]

roc<-roc(d$y~d$x, auc = T); return(roc$auc)

}

for (j in 1:4){

x<-roc.sbp.k16[[j]]$original.predictor

y<-roc.sbp.k16[[j]]$original.response

m<-data.frame(x, y)

boot<-boot(m, x="x", y="y", R=2000, statistic=fx)

boot.auc.sbp.k16[,j]<-boot$t

bootraw.auc.sbp.k16[[j]]<-boot

}

With these bootstrap variations, we compared the C-statistics and CCRs among the *K* values and the SD levels and evaluated the interactions between the *K*s and the SD levels using mixed linear effect models. In the models, each combination of the *K*s and SD levels was used as an identifier, variations within the combination were considered random effects, and the variations among the *K* values and the SD levels were considered fixed effects. All bootstrap variations of C-statistics and CCRs are provided in Supplementary Data S3.

5. Estimation of mean SBP and DBP using high BP count.

Linear regression models were used to predict the mean SBPs and DBPs corresponding to the high SBP and DBP counts with 95% prediction intervals (PIs). A linear regression model was produced for each SD level at *K* = 24.

### Validation

Validation for the count-based decision method for uncontrolled BP was performed using HBPM data obtained from 424 patients who had visited the outpatient clinic of the cardiology department for antihypertensive medications at Hanyang University Seoul Hospital from November 2017 to September 2018. The HBPM data collection and study protocol adhered to the Declaration of Helsinki. The use of these HBPM data was approved by the Institutional Review Board of Hanyang University Seoul Hospital, and informed consent was waived because the HBPM data were retrospectively obtained from the electrical medical records. BP measurements on the first day of HBPM were not included to improve the reproducibility of the HBPM data^12^. HBPM data from 12 patients whose number of BP readings was < 8 were excluded. Patients were categorized into 3 groups according to *K*s (8–15 times, 16–23 times, and ≥ 24 times), SDs (< 10 mmHg, 10–14.9 mmHg, and ≥ 15 mmHg), SBP ranges (< 40 mmHg, 40–59 mmHg, and ≥ 60 mmHg), and mean SBPs/DBPs.

Diagnostic performances of the count-based decision method for uncontrolled BP were assessed using ROC curve analyses and C-statistics. Because, unlike *K*s with 3 fixed levels in the simulation cohort, the numbers of actual HBP readings widely varied patient by patient from 8 to 70, we used the ratio between the high BP count (C) and the number of total HBP readings (T) (abbreviated further as the C/T ratio), instead of the high BP counts, to estimate C-statistics for uncontrolled BP. Other diagnostic performance indices, including sensitivity, specificity, PPV, NPV, and CCR, were assessed when the C/T ratio was ≥ 0.5, as follows:

*# Calculation of C/T ratio*

*## “V” is a list of matrices containing columns for home SBPs and DBPs*

*## “m” is a dataframe containing columns for the mean SBPs and mean DBPs*

*## “nmeasure” indicates the number of total BP readings*

*## “rate.sbp135” and “rate.dbp85” indicate the systolic and diastolic C/T ratios, respectively.*

for(i in 1:NROW(V)){

dt<-V[[i]]

m$n.sbp135[i]<-NROW(which(dt$sbp >=135, 1, 0))

m$n.dbp85[i]<-NROW(which(dt$dbp >=85, 1, 0))

}

m$rate.sbp135<-m$n.sbp135/m$nmeasure

m$rate.dbp85<-m$n.dbp85/m$nmeasure

*# ROC curve analysis between uncontrolled SBP (or DBP) and systolic (or diastolic) C/T ratio*

*## “sys.htn” indicates uncontrolled SBP (mean SBP≥135 mmHg)*

*## “dia.htn” indicates uncontrolled DBP (mean DBP≥85 mmHg)*

roc.sbp.rate<-roc(sys.htn~crate.sbp135, data=m, ciT, auc=T)

roc.dbp.rate<-roc(dia.htn~rate.dbp85, data=m, ci=T, auc=T)

*# Calculation of sensitivity, specificity, PPV, NPV and CCR at a C/T ratio* ≥ *0.5*

*## “n.sbp135.bi” indicates a binary variable of the systolic C/T ratio dichotomized at 0.5*

*## “n.dbp85.bi” indicates a binary variable of the diastolic C/T ratio dichotomized at 0.5*

*## “roc.n.sbp” and “roc.n.dbp” indicate ROC curve object at the C/T ratio* ≥ *0.5*

*## “coordx.n.sbp” and “coordx.n.dbp” indicate the diagnostic performance indices*

ret<-c("threshold", "sens", "spec", "ppv", "npv", "accuracy")

m$n.sbp135.bi<-ifelse(m$rate.sbp135>=0.5, 1, 0)

roc.n.sbp<-roc(sys.htn~n.sbp135.bi, data=m, ci=T, auc=T)

coordx.n.sbp<-coords(roc.n.sbp, ret=ret)

m$n.dbp85.bi<-ifelse(m$rate.dbp85>=0.5, 1, 0)

roc.n.dbp<-roc(dia.htn~n.dbp85.bi, data=m, ci=T, auc=T)

coordx.n.dbp<-coords(roc.n.dbp, ret=ret)

Mean HBPs were predicted with the 95% PIs using the C/T ratio. Unlike the simulated HBPs generated within the programmed range, the real HBPs had a wider distribution ranging between 89 and 183 mmHg for the mean SBPs and 59–125 mmHg for the mean DBPs. Consequently, the fitting curve between the high BP counts and the mean BP was expected to resemble a logit curve (y = *C* + *b *× log(x/(1-x))|0 < x < 1). However, because the fitting curve will be linear within the range applied to the simulated HBPs, we fitted the C/T ratios to the mean HBPs using a linear curve within the range of the simulated HBPs whose SD was most similar to the SD of the real HBPs, in addition to the logit curve.

### Statistical analysis

All simulations and statistical analyses were performed using R-4.04 and RStudio-1.3. The sample size of 100 cases was determined empirically, given that a physician would prescribe 50 patients per session and 100 patients per day in primary care clinic settings. To assess the diagnostic performances of the count-based decision method for uncontrolled BP, ROC curve analysis with the “pROC” package was used. The 95% CIs of the C-statistics were estimated using 2 methods, Delong’s method and the bootstrap resampling method. The comparisons of C-statistics and CCRs among *K*s and SDs were performed through mixed linear effect models on the 24,000 permuted results of the C-statistics and CCRs from the bootstrap resamples using the “lme4” package. A linear regression model in the “stats” package was used to fit the high BP counts and the mean BPs on both a linear curve and a logit curve. C-statistics of < 0.70, 0.70–0.89, and > 0.90 were interpreted as poor, moderate, and high in diagnostic accuracy, and a *p* value < 0.05 was considered to be significant.

## Results

### Simulated HBPs

Because, in the simulated HBP cohort, there were 12 possible combinations between the 3 K values (16, 20, and 24) and the 4 SD levels (5, 10, 15, and 20), 1200 cases of HBPM series were generated.

In the simulated HBP cohort, the C-statistics of high SBP and DBP counts for uncontrolled SBP and DBP at all combinations of *K*s and SDs were higher than 0.9 (Table 1), except that the C-statistic at a *K* of 24 and an SD of 20 was 0.868 (95% CI 0.803–0.934). In general, C-statistics were higher when SD levels were lower, while they did not appear to be different as *K* values changed (Table 1). The best thresholds of high BP counts were ≥ 8 or 9 for a *K* of 16, ≥ 10 or 11 for a *K* of 20, and ≥ 11–13 for a *K* of 24, depending on the corresponding SD levels, which were all near half of *K*s (Supplementary Table S1). At the best thresholds, most sensitivities, specificities, PPVs, NPVs, and CCRs were > 0.8, although, in general, the diagnostic performance indices decreased as SD levels increased (Table 1 and Supplementary Table S1).

**Table 1:** C-statistics of high BP counts for uncontrolled BP.

*K*	SBP		DBP	
	SD	C-statistics	SD	C-statistics
24	5	0.970 (0.945–0.996)	3.5	1.000 (1.000–1.000)
	10	0.933 (0.891–0.976)	7	0.983 (0.964–1.000)
	15	0.933 (0.887–0.979)	10.5	0.974 (0.950–0.998)
	20	0.868 (0.803–0.934)	14	0.966 (0.938–0.994)
20	5	0.984 (0.968–1.000)	3.5	0.996 (0.991–1.000)
	10	0.961 (0.932–0.991)	7	0.988 (0.971–1.000)
	15	0.933 (0.890–0.976)	10.5	0.986 (0.969–1.000)
	20	0.919 (0.869–0.968)	14	0.948 (0.911–0.985)
16	5	0.973 (0.945–1.000)	3.5	0.998 (0.993–1.000)
	10	0.944 (0.903–0.986)	7	0.978 (0.959–0.998)
	15	0.954 (0.922–0.988)	10.5	0.965 (0.938–0.992)
	20	0.920 (0.871–0.969)	14	0.911 (0.855–0.967)

*K* number of BP measurements, *SD* standard deviation, *SBP* systolic blood pressure, *DBP* diastolic blood pressure.

When the threshold was set to 8, 10, and 12, half of *K*s, most diagnostic performance indices remained similarly high as those at the best thresholds, except that CCR for uncontrolled SBPs at a *K* of 24 and an SD of 20 mmHg was 0.76 (Table 2). Similar to the results at the best thresholds, the diagnostic performance indices at half of *K*s decreased as SD levels increased, while they did not appear to be different as *K*s changed.

**Table 2:** Diagnostic performance of the count-based decision method at half of *K*s for uncontrolled BP in the simulated cohort.

*K*	SBP							DBP						
	SD	Uncontrolled SBP (N = 100)	Sensitivity	Specificity	PPV	NPV	CCR	SD	Uncontrolled DBP (N = 100)	Sensitivity	Specificity	PPV	NPV	CCR
24 threshold ≥ 12	5	50	0.907	0.895	0.867	0.927	0.90	3.5	54	1.000	1.000	1.000	1.000	1.00
	10	50	0.880	0.800	0.815	0.870	0.84	7	56	1.000	0.867	0.902	1.000	0.94
	15	49	0.878	0.902	0.896	0.885	0.89	10.5	57	0.912	0.930	0.945	0.889	0.92
	20	48	0.854	0.673	0.707	0.833	0.76	14	53	0.962	0.766	0.823	0.947	0.87
20 threshold ≥ 10	5	44	0.980	0.882	0.889	0.978	0.93	3.5	51	0.980	0.959	0.962	0.979	0.97
	10	54	0.918	0.902	0.9	0.92	0.91	7	47	0.957	0.906	0.9	0.96	0.93
	15	49	0.860	0.780	0.796	0.848	0.82	10.5	53	0.943	0.957	0.962	0.938	0.95
	20	50	0.907	0.719	0.709	0.911	0.80	14	54	0.889	0.848	0.873	0.867	0.87
16 threshold ≥ 8	5	48	0.958	0.904	0.902	0.959	0.93	3.5	51	0.98	0.939	0.943	0.979	0.96
	10	50	0.918	0.902	0.900	0.920	0.88	7	53	0.962	0.830	0.864	0.951	0.90
	15	54	0.907	0.761	0.817	0.875	0.84	10.5	56	0.929	0.841	0.881	0.902	0.89
	20	51	0.863	0.816	0.830	0.851	0.84	14	53	0.943	0.766	0.82	0.923	0.86

*K* number of BP measurements, *SBP* systolic blood pressure, *DBP* diastolic blood pressure, *PPV* positive predictive value, *NPV* negative predictive value, *CCR* correct classification rate.

Uncontrolled SBP/DBP were defined as the mean SBP ≥ 135 mmHg or the mean DBP ≥ 85 mmHg.

**Figure 1:**
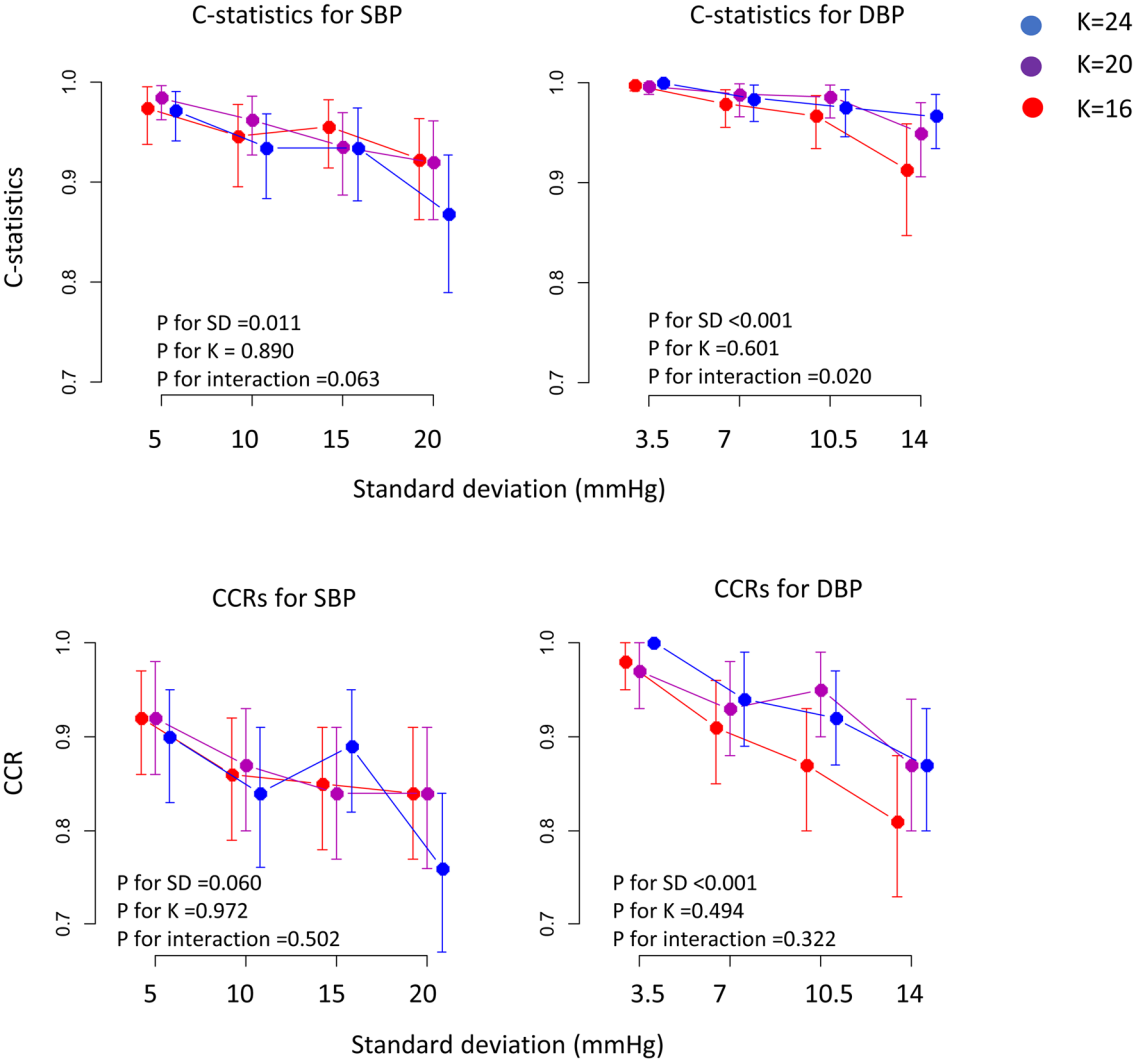
Diagnostic performances according to *K*s and SDs in the simulated HBP cohort. C-statistics and CCRs for both SBP and DBP gradually decreased as SDs increased. The influences of *K*s on both the C-statistics and CCRs were not statistically significant. Each CI was generated using a set of 2000 bootstrap samples from the original simulated HBP data, and the *p* values were generated using mixed-linear effect models. *SD* standard deviation, *K* number of measurements.

Mixed linear effect models using the bootstrap variations of the diagnostic performance indices showed that the C-statistics of high BP counts for uncontrolled SBPs and DBPs decreased as SDs increased, while they did not significantly differ among *K*s (Fig. 1). The decreases in C-statistics for uncontrolled DBPs were stiffer as *K*s decreased. Similar to the C-statistics, CCRs for uncontrolled SBPs and DBPs decreased as SDs increased, while they did not change with *K*s. In general, C-statistics for uncontrolled DBPs were higher than C-statistics for uncontrolled SBPs (median [interquartile range] 0.982 [0.964–0.995] vs. 0.947 [0.922–0.969] for C-statistics, and 0.93 [0.88–0.97] vs. 0.87 [0.83–0.90] for CCRs; *p* < 0.001 for both in a Mann–Whitney test).

**Figure 2:**
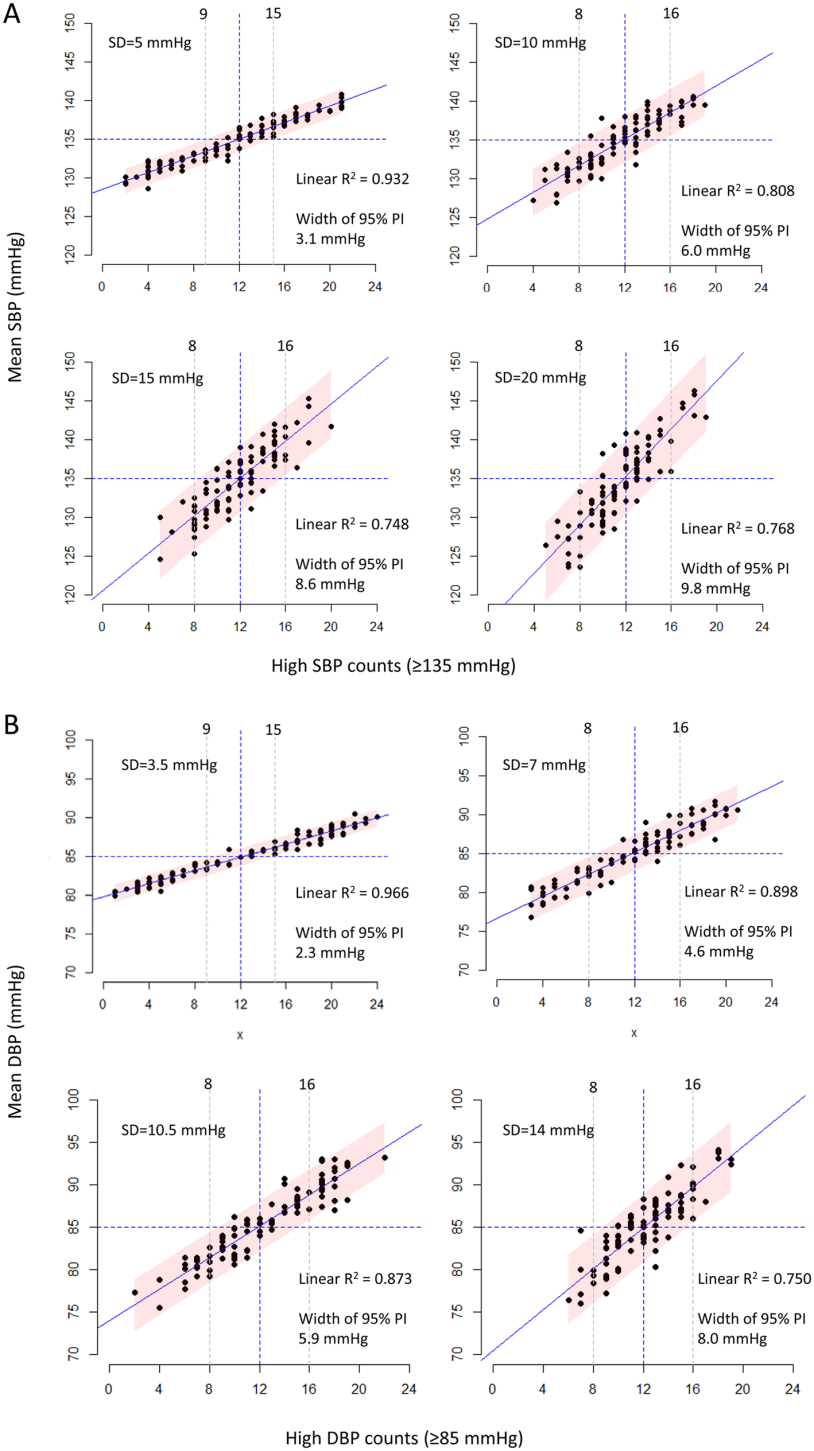
Relationship between high BP counts and mean SBP/DBP in the simulated HBP cohort (when *K* = 24; panel **A** for SBP and panel **B** for DBP). There were tight linear associations between high BP counts and mean SBP/DBP. As SDs increased, the strength of the association decreased, and the width of the 95% PIs increased. When the SD was 5, high BP counts ≤ 9 indicated controlled SBP/DBP, and high BP counts ≥ 15 indicated uncontrolled SBP/DBP with ≥ 95% confidence. When SD > 5, high BP counts ≤ 8 indicated controlled SBP/DBP, and high BP counts ≥ 16 indicated uncontrolled SBP/DBP with ≥ 95% confidence. Each dot represents a series of 24 simulated HBPs, and the dots in a panel represent 100 series of simulated HBPs at an SD level. The blue solid lines indicate the linear regression fits between high BP counts and mean SBP/DBP, and the red ribbons indicate the 95% PI of the mean SBP/DBP. The gray dotted lines indicate the smallest and the largest high BP counts outside the upper and lower limits of the 95% PIs, respectively. High SBP/DBP was defined as an SBP ≥ 135 mmHg/DBP ≥ 85 mmHg. *PI* prediction interval, *SBP* systolic blood pressure, *DBP* diastolic blood pressure, *SD* standard deviation.

Linear regression models showed that high SBP/DBP counts were linearly associated with mean SBP/DBP, while the association strengths decreased and the 95% PI widths of the linear models increased for both SBP and DBP as SDs increased (Fig. 2A for SBP; Fig. 2B for DBP). For an SD of 5 mmHg (3.5 mmHg for DBPs), high BP counts ≥ 15 (62.5%), and ≤ 9 (37.5%) of the 24 simulated HBP readings predicted uncontrolled BP and controlled BP, respectively, with ≥ 95% confidence (gray dotted lines, Fig. 2A). For the other SDs, high BP counts ≥ 16 (66.7%) and ≤ 8 (33.3%) of the 24 simulated HBP readings predicted uncontrolled BP and controlled BP, respectively, with ≥ 95% confidence (gray dotted lines, Fig. 2B).

### Validation

After the exclusion of 12 patients with < 8 HBP readings, HBPM data from 412 patients on antihypertensive medications were analyzed to validate the count-based decision method. All HBPM data used in the validation procedure are provided in Supplementary Data S4 and S5. In the validation cohort, the age was 58.4 ± 12.1 years, and 217 patients (52.7%) were females. Body mass index (BMI) was 25.4 ± 13.2 kg/m^2^, and diabetes was found in 47 patients (11.4%). The mean SBP was 128.2 ± 14.8 mmHg, and the mean DBP was 79.9 ± 9.7 mmHg. Uncontrolled BP (≥ 135/85 mmHg) was prevalent in 40.3% (n = 166). Detailed characteristics of the patients are described in Table 3.

**Table 3:** Characteristics of patients in the validation cohort.

Characteristics	Values
N	412
Age (years)	58.4 ± 12.1
Female	217 (52.7%)
BMI (kg/m^2^)	25.4 ± 13.2
Diabetes mellitus	47 (11.4%)
Mean SBP (mmHg)	128.2 ± 14.8
Mean DBP (mmHg)	79.9 ± 9.7
SBP range (mmHg)	89.1–182.8
DBP range (mmHg)	52.0–125.4
Uncontrolled BP (≥ 135/85 mmHg)	166 (40.3%)
Uncontrolled SBP (≥ 135 mmHg)	115 (27.9%)
Uncontrolled DBP (≥ 85 mmHg)	140 (29.1%)
SBP between 130–140 mmHg	108 (26.2%)
DBP between 80–90 mmHg	147 (35.7%)

*BMI* body mass index, *SBP* systolic blood pressure, *DBP* diastolic blood pressure.

The overall C-statistic of the C/T ratio was 0.986 (95% CI 0.975–997) for uncontrolled SBP and 0.974 (95% CI 0.961–0.987) for uncontrolled DBP (Table 4). The CCR at a C/T ratio ≥ 0.5 was 0.954 for uncontrolled SBP and 0.925 for uncontrolled DBP. The C-statistics of the C/T ratios were highest in the group with *K*s between 16 and 24, that with the lowest SDs (< 10 mmHg for uncontrolled SBP, < 7 mmHg for uncontrolled DBP) and that with the lowest BP differences (< 40 mmHg for uncontrolled SBP, < 30 mmHg for uncontrolled DBP) for both uncontrolled SBP and DBP (Table 4). CCRs were > 0.90 in all categories except in the group with *K*s ≥ 24 times, SD ≥ 10 mmHg, and BP difference ≥ 45 mmHg for uncontrolled DBP. In the groups with a mean SBP between 130 and 140 mmHg and those with a mean DBP between 80 and 90 mmHg, C-statistics and all diagnostic performance indices, including sensitivity, specificity, PPV, NPV, and CCR, were lower than those in all patients but remained > 0.75 (Table 4).

**Table 4:** Diagnostic performances of high BP counts for uncontrolled BP in the validation cohort.

			N	Uncontrolledness	Sensitivity	Specificity	PPV	NPV	CCR	C-statistics of C/T ratio*
SBP	Overall		412	115 (27.9%)	0.939	0.960	0.900	0.976	0.954	0.986 (0.939–0.997)
	*K*s	8–15	59	19 (32.2%)	0.947	0.950	0.900	0.974	0.949	0.978 (0.945–1.000)
		16–23	233	59 (25.3%)	0.967	0.954	0.879	0.988	0.957	0.997 (0.993–1.000)
		≥ 24	120	37 (30.8%)	0.892	0.976	0.943	0.953	0.950	0.963 (0.923–1.000)
	SDs of SBP	< 10 mmHg	200	36 (18.0%)	1.000	0.988	0.947	1.000	0.990	0.999 (0.997–1.000)
		10–14.9 mmHg	139	42 (30.2%)	0.881	0.918	0.822	0.947	0.906	0.960 (0.920–0.999)
		≥ 15 mmHg	73	37 (50.7%)	0.946	0.944	0.946	0.944	0.945	0.975 (0.941–0.999)
	Maximum ∆SBP	< 40 mmHg	200	33 (16.5%)	0.970	0.982	0.914	0.994	0.980	0.991 (0.974–1.000)
		40–59 mmHg	145	52 (35.9%)	0.942	0.925	0.875	0.966	0.931	0.979 (0.955–1.000)
		≥ 60 mmHg	67	30 (44.8%)	0.900	0.946	0.931	0.921	0.925	0.980 (0.950–1.000)
	Mean SBP	130–139 mmHg	178	43 (39.8%)	0.860	0.831	0.771	0.900	0.843	0.892 (0.820–0.965)
DBP	Overall		412	120 (29.1%)	0.867	0.949	0.874	0.945	0.925	0.974 (0.961–0.987)
	*K*s	8–15	59	20 (33.9%)	0.950	0.897	0.826	0.972	0.915	0.967 (0.931–1.000)
		16–23	233	64 (27.5%)	0.922	0.953	0.881	0.970	0.944	0.989 (0.980–0.998)
		≥ 24	120	36 (30.0%)	0.722	0.964	0.897	0.890	0.892	0.952 (0.918–0.986)
	SDs of DBP	< 7 mmHg	200	53 (22.7%)	0.868	0.978	0.920	0.962	0.953	0.985 (0.973–0.997)
		7–9.9 mmHg	139	30 (28.8%)	0.900	0.905	0.794	0.957	0.904	0.971 (0.941–1.000)
		≥ 10 mmHg	73	37 (49.3%)	0.838	0.895	0.886	0.850	0.867	0.942 (0.894–0.989)
	Maximum ∆DBP	< 30 mmHg	200	70 (25.6%)	0.871	0.966	0.897	0.956	0.941	0.982 (0.970–0.995)
		30–44 mmHg	145	19 (24.1%)	0.895	0.917	0.773	0.965	0.911	0.976 (0.949–1.000)
		≥ 45 mmHg	67	31 (51.7%)	0.839	0.897	0.897	0.839	0.867	0.937 (0.880–0.994)
	Mean DBP	80–89 mmHg	147	62 (42.2%)	0.790	0.824	0.766	0.843	0.810	0.858 (0.794–0.923)

*K* number of HBP measurements, *∆SBP/DBP* difference in SBP/DBP, *SD* standard deviation, *SBP* systolic blood pressure, *DBP* diastolic blood pressure, *C/T ratio* ratio between the high BP count (C) and the number of total HBP readings (T), *CCR* correct classification rate, *PPV* positive predictive value, *NPV* negative predictive value.

The high SBP/DBP were defined as SBP ≥ 135 mmHg or DBP ≥ 85 mmHg.

**Figure 3:**
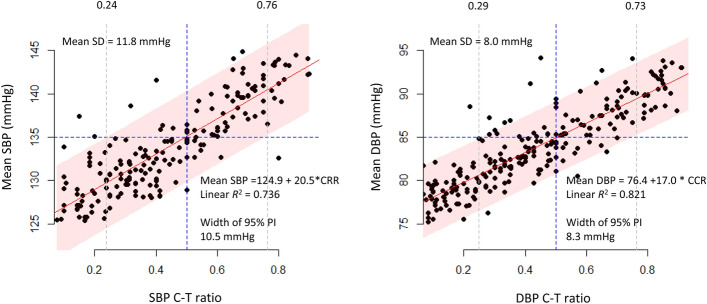
Relationship between the C/T ratio and the mean SBP/DBP in the validation cohort. There was a strong linear association between the C/T ratio and the mean SBP/DBP. Because the SDs of the mean SBP/DBP were 11.8 mmHg and 8.0 mmHg, respectively, the linear regression models between the C/T ratio and mean BP were produced within the mean SBP of 125–145 mmHg and the mean DBP of 75–95 mmHg, which are similar to those of the simulated SBPs with an SD of 10 mmHg and that of the simulated DBPs with an SD of 7 mmHg. High SBP/DBP was defined as SBP ≥ 135 mmHg/DBP ≥ 85 mmHg. Each dot represents a real HBPM series from a patient. The blue solid lines represent the linear regression fits, and the red ribbons represent the 95% PIs of the mean SBP/DBP. The gray dotted lines represent the C/T ratios at the upper and lower limits of the 95% PIs. *C/T ratio* ratio between the high BP count (C) and the number of total HBP readings (T), *PIs* prediction intervals, *SBP* systolic blood pressure, *DBP* diastolic blood pressure, *SD* standard deviation.

Both mean SBP and DBP were highly associated with the corresponding C/T ratios (Fig. 3 and Supplementary Fig. S1). The association between mean SBP/DBP and the corresponding C/T ratios fit well in logit curves in the entire SBP and DBP ranges (Supplementary Fig. S1). However, because the mean SDs of SBP and DBP were 11.8 mmHg and 8.0 mmHg, we produced linear models within a range of the mean SBPs (125–145 mmHg) and the mean DBP (75–95 mmHg), similar to the range used in the simulated cohort, where the SDs of SBP and DBP were 10 mmHg and 7 mmHg, respectively. The linear models showed that a C/T ratio < 0.24 indicated controlled SBP, that a C/T ratio > 0.76 indicated uncontrolled SBP with ≥ 95% confidence, and that a C/T ratio < 0.29 indicated controlled DBP and a C/T ratio > 0.73 indicated uncontrolled DBP with ≥ 95% confidence (gray dotted lines in Fig. 3, Table 5). The average widths of the 95% PIs derived within the BP ranges were 10.5 mmHg for SBP and 8.3 mmHg for DBP.

**Table 5:** Estimated mean SBP/DBP according to the C/T ratio.

Model summary	Rate	Mean SBP		Mean DBP	
		Estimates	95% Prediction interval	Estimates	95% Prediction interval
Logit model	0.2	126.5	117.3–135.6	79.7	72.9–86.6
Y = *C* + *β *× log(X/(1 − X))|0 < X < 1	0.3	130	120.8–139.1	81.9	75.1–88.7
Entire SBP/DBP ranges	0.4	132.8	123.7–141.9	83.7	76.9–90.5
	0.5	135.4	126.3–144.6	85.3	78.5–92.2
	0.6	138	128.9–147.2	87	80.2–93.8
	0.7	140.9	131.7–150.0	88.8	81.9–95.6
	0.8	144.4	135.2–153.5	91	84.1–97.8
Linear model	0.2	128.9	123.7–134.2	79.8	75.6–84.0
Y = β × X + C	0.3	131	125.7–136.2	81.5	77.4–85.7
SBP 125–145 mmHg	0.4	133	127.8–138.3	83.2	79.1–87.4
DBP 75–95 mmHg	0.5	135.1	129.8–140.3	84.9	80.8–89.1
	0.6	137.1	131.9–142.4	86.6	82.5–90.8
	0.7	139.2	133.9–144.4	88.3	84.2–92.5
	0.8	141.2	135.9–146.5	90	85.8–94.2

*C/T ratio* ratio between the high BP count (C) and the number of total HBP readings (T), *SBP* systolic blood pressure, *DBP* diastolic blood pressure.

A high SBP/DBP was defined as SBP ≥ 135 mmHg or DBP ≥ 85 mmHg.

## Discussion

In this study, we developed a quick interpretation method to determine whether BP was controlled using the high BP counts in simulated HBPs and validated the method using real HBPM data from patients under antihypertensive medications. A high BP count ≥ half of *K*s was highly accurate in the diagnosis of uncontrolled BP. The accuracy of the high BP counts was highly dependent on the variations of BPs but was not affected by *K*s within the simulated range, 16–24. In the validation, a C/T ratio ≥ 0.5 accurately diagnosed uncontrolled BP, a C/T ratio was highly correlated with the mean HBPs, and a C/T ratio ≤ 0.2 or ≥ 0.8 strongly indicated control or lack of control of BP, respectively, with > 95% confidence. In these analysis results from the real HBPM data, we found that the decision method using the C/T ratio could allow physicians to (1) accurately diagnose uncontrolled BP, (2) estimate the approximate mean BP, and (3) rapidly rule in or rule out uncontrolled BP with high confidence in a patient with HBPM records.

Although HBPM is recommended for the diagnosis of hypertension and titration of BP in guidelines^13^ and has many advantages over office BP^1^, the use of HBPM remains challenging because many HBP devices were not validated, were expensive to low-income families and did not provide friendly interfaces to review HBP records easily^14^. The use of HBP as the target of BP control is especially difficult in elderly patients because keeping HBP records requires a real commitment and keenness^10^. A mean HBP ≥ 135/85 mmHg has been recommended as a criterion for uncontrolled HBP^1^, which may also hamper the wider use of HBPM because calculating mean HBPs is burdensome not only for patients but also for physicians unless clinics are sufficiently resourced. In this regard, an intuitive tool to quickly assess the controlledness of HBP is desired, and the current study is the first to propose the use of a count-based method to decide whether HBP is controlled.

The validity of the count-based decision method was tested using real HBPM data. Because the accuracy of the count-based decision method depends on the distributions of BPs, other real-world HBP readings with SDs similar to those in the validation dataset including the Asian BP at Home Study data^15^ may show similarly high diagnostic performances for uncontrolled BP. Although the accuracy of the count-based decision method was blunted when the mean SBP/DBP was restricted to 130–140/80–90 mmHg (around the criterion for uncontrolled HBP, 135/85 mmHg) in which it would be most difficult for a physician to recognize the lack of control of BP at a glance to a series of HBP readings, the C-statistics remained > 0.85, and other diagnostic performance indices, including CCR, at the C/T ratio ≥ 0.5 also remained substantially high.

In the results, we also showed that the C/T ratios were highly correlated with the mean SBP/DBP. Although the relationships between the C/T ratio and mean BP must resemble logit curves, the relationship between the C/T ratio and mean BP was similarly well explained by the linear models within the mean BP ranges close to 135/85 mmHg. Using its close correlation with mean BP, the C/T ratio could provide a quick approximation of the mean BP. If a physician could count the number of high SBPs in the last 10 or 20 HBP readings, the physician may be able to obtain an estimate of the mean BP using the table of the C/T ratios and mean BP estimates, as shown in Table 5. The C/T ratio could be useful for a physician to rapidly rule in or rule out uncontrolled SBP/DBP because C/T ratios ≤ 0.2 and ≥ 0.8 indicated the control and lack of control of BPs, respectively, with ≥ 95% confidence. This rule-in/rule-out method for uncontrolled BP may be effective in crowded clinics where physicians have to make rapid decisions regarding the controlledness of BP.

There are several limitations in our study. First, because the simulated HBPs were produced using a function generating random numbers with a normal distribution, the simulated HBPs would not harbor any characteristics related to the diurnal circadian and weekly rhythm potentially residing in a series of real HBP readings^16^, which may explain the difference between the accuracy of the count-based decision method in the simulated HBPs and that in the real HBPM data. Although SDs were similar, the skewness and kurtosis may be greater in the real HBP readings because of these diurnal and weekly regularities, which may blunt the accuracy of the decision method. Second, we simulated HBPs with *K*s between 16 and 24. These *K* values were the most frequent numbers of measurements in our real HBPM data. The accuracy of the decision method appeared to be independent of *K* values within this range. However, guidelines recommend the numbers of HBP measurements as twice a day for 3–7 days (6–14 times)^1,3^. At higher *K* values, the accuracy of the count-based decision method would converge into one value, but the accuracy may vary more widely at lower *K* values. Third, the real HBP readings used in validation were obtained from patients on antihypertensive medications. The prevalence of a mean BP ≥ 135/85 mmHg and the levels of interest and education regarding HBPM may be different in those not diagnosed with hypertension and taking any antihypertensive agents. Therefore, the accuracy of the decision method using the C/T ratio ≥ 0.5 should be cautiously interpreted in other situations where HBPM is recommended.

Although HBPM is effective in identifying white-coat phenomena, masked (uncontrolled) hypertension and nocturnal hypertension^2^, the accuracy of HBPM in the diagnosis of hypertension remains insufficient to replace ambulatory BP monitoring with a sensitivity of 86% and a specificity of 62%^17,18^. Because the count-based decision method was developed using the HBPM series, it is desirable to evaluate its diagnostic accuracy for uncontrolled hypertension compared to the diagnostic accuracy using ambulatory BP monitoring.

In conclusion, the count-based decision method using a C/T ratio ≥ 0.5 could provide a quick and accurate assessment of whether the target BP was achieved in a series of HBPM. When the SDs of SBPs/DBPs are within a usual range, the C/T ratio could also allow a physician to estimate the approximate of the mean HBPs and to rapidly rule in or rule out the lack of control of BPs. The C/T ratio could be helpful for physicians trying to use HBPM as their primary measure for target BP achievement to quickly assess the controlledness of HBP.

## Supplementary Information

Supplementary Information 1.

Supplementary Information 2.

Supplementary Information 3.

Supplementary Information 4.

Supplementary Information 5.

## Data Availability

All data generated or analyzed during this study are included in this published article and its Supplementary Information files. The raw datasets, including the simulated HBPs, the bootstrap resampling results for C-statistics and CCRs, the real HBPs, and the R script used to simulate and analyze the data, are all provided in Supplementary Data S1–S5.
