# Supplementary material for: A count-based decision method for target blood pressure achievement in home blood pressure monitoring data interpretation for clinical practices
Source: Sci Rep. 2022 Mar 10;12:3897. doi: 10.1038/s41598-022-04913-9 (PMC8913602; doi:10.1038/s41598-022-04913-9)
Supplement: Supplementary file 1 — Supplementary Information 1. [file 41598_2022_4913_MOESM1_ESM.docx]

Supplementary Data

Supplementary data 1

R codes (written in RStudio 1.3)

*For the simulation cohort*

library(pROC)

library(boot)

library(erer)

library(lme4)

library(car)

###########################################################################################

### mean SBP, DBP generation

set.seed(195377)

N<-100

M.sbp<-runif(n=N, min = 130, max = 140)

M.dbp<-runif(n=N, min = 80, max = 90)

hist(M.sbp); summary(M.sbp)

hist(M.dbp); summary(M.dbp)

###########################################################################################

### K=16 individual SBP DBP generation

N<-100

K<-c(16, 20, 24)

SD<-c(5, 10, 15, 20)

sd_label<-c("SD=5", "SD=10", "SD=15", "SD=20")

k_label<-c("k=16", "k=20", "k=24")

id<-c(paste0("Sim", "0", 0:9), paste0("Sim", 10:99))

dimnames<-list(id, sd_label)

msd<-matrix(NA, ncol=NROW(SD), nrow=N, dimnames = dimnames)

sbp.all<-list(NA, NA, NA); names(sbp.all)<-k_label

dbp.all<-list(NA, NA, NA); names(dbp.all)<-k_label

bp.list.all<-list(NA, NA, NA); names(bp.list.all)<-k_label

set.seed(195377)

for (h in 1:3) {

mk<-matrix (NA, ncol = K[h], nrow = N)

sbp<-list(mk, mk, mk, mk)

dbp<-list(mk, mk, mk, mk)

names(sbp)<-sd_label

names(dbp)<-sd_label

meansbp<-msd

meandbp<-msd

shtn<-msd

dhtn<-msd

nsbp135<-msd

ndbp85<-msd

for(j in 1:NROW(SD)){

for(i in 1:N){

sbp[[j]][i,]<-rnorm(n=K[h], mean = M.sbp[i], sd=SD[j])

dbp[[j]][i,]<-rnorm(n=K[h], mean = M.dbp[i], sd=SD[j]*0.7)

nsbp135[i,j]<-NROW(which(sbp[[j]][i,]>=135))

ndbp85[i,j]<-NROW(which(dbp[[j]][i,]>=85))

}

meansbp[,j]<-apply(sbp[[j]], 1, mean)

meandbp[,j]<-apply(dbp[[j]], 1, mean)

shtn[,j]<-ifelse(meansbp[,j]>=135, 1, 0)

dhtn[,j]<-ifelse(meandbp[,j]>=85, 1, 0)

}

bp.list<-list(meansbp, meandbp, shtn, dhtn, nsbp135, ndbp85)

names(bp.list)<-c("meansbp", "meandbp", "shtn", "dhtn", "nsbp135", "ndbp85")

sbp.all[[h]]<-sbp

dbp.all[[h]]<-dbp

bp.list.all[[h]]<-bp.list

}

write.list(sbp.all[[1]], file = "SBP_K16.csv")

write.list(sbp.all[[2]], file = "SBP_K20.csv")

write.list(sbp.all[[3]], file = "SBP_K24.csv")

write.list(dbp.all[[1]], file = "DBP_K16.csv")

write.list(dbp.all[[2]], file = "DBP_K20.csv")

write.list(dbp.all[[3]], file = "DBP_K24.csv")

write.list(bp.list.all[[1]], file = "BP_list_K16.csv")

write.list(bp.list.all[[2]], file = "BP_list_k20.csv")

write.list(bp.list.all[[3]], file = "BP_list_k24.csv")

sbp.k16<-sbp.all[[1]]; sbp.k20<-sbp.all[[2]]; sbp.k24<-sbp.all[[3]]

dbp.k16<-dbp.all[[1]]; dbp.k20<-dbp.all[[2]]; dbp.k24<-dbp.all[[3]]

bp.list.k16<-bp.list.all[[1]]; bp.list.k20<-bp.list.all[[2]]; bp.list.k24<-bp.list.all[[3]]

#########################################################################################

##ROC, Bootstrapping data generation

set.seed(195377)

roc.sbp.all<-list(NA, NA, NA); names(roc.sbp.all)<-k_label

bootraw.auc.sbp.all<-list(NA, NA, NA); names(bootraw.auc.sbp.all)<-k_label

boot.auc.sbp.all<-list(NA, NA, NA); names(boot.auc.sbp.all)<-k_label

bootraw.ccr.sbp.all<-list(NA, NA, NA); names(bootraw.ccr.sbp.all)<-k_label

boot.ccr.sbp.all<-list(NA, NA, NA); names(boot.ccr.sbp.all)<-k_label

roc.dbp.all<-list(NA, NA, NA); names(roc.sbp.all)<-k_label

bootraw.auc.dbp.all<-list(NA, NA, NA); names(bootraw.auc.dbp.all)<-k_label

boot.auc.dbp.all<-list(NA, NA, NA); names(boot.auc.dbp.all)<-k_label

bootraw.ccr.dbp.all<-list(NA, NA, NA); names(bootraw.ccr.dbp.all)<-k_label

boot.ccr.dbp.all<-list(NA, NA, NA); names(boot.ccr.dbp.all)<-k_label

for (h in 1:3) {

roc.sbp <-list(NA, NA, NA, NA)

bootraw.auc.sbp<-list(NA, NA, NA, NA)

boot.auc.sbp<-matrix(NA, nrow = 2000, ncol = 4)

bootraw.ccr.sbp<-list(NA, NA, NA, NA)

boot.ccr.sbp<-matrix(NA, nrow = 2000, ncol = 4)

roc.dbp<- list(NA, NA, NA, NA)

bootraw.auc.dbp<-list(NA, NA, NA, NA)

boot.auc.dbp<-matrix(NA, nrow = 2000, ncol = 4)

bootraw.ccr.dbp<-list(NA, NA, NA, NA)

boot.ccr.dbp<-matrix(NA, nrow = 2000, ncol = 4)

fx.auc<-function(data, indices, x, y){

d<-data[indices, ]

roc<-roc(d$y~d$x, auc=T); return(roc$auc)

}

k<-(K[h]-1)/2

fx.ccr<-function(data, indices, x, y){

d<-data[indices, ]

roc<-roc(d$y~d$x, auc=T)

coords<-coords(roc, ret=c("thres","accuracy"))

out<-coords$accuracy[coords$threshold==k]

return(ifelse(NROW(out)>0, out, 1))

}

dl<-bp.list.all[[h]]

for (i in 1:4) {

#ROC model

roc.sbp[[i]]<-roc(dl$shtn[,i]~dl$nsbp135[,i], ci=T, auc=T)

roc.dbp[[i]]<-roc(dl$dhtn[,i]~dl$ndbp85[,i], ci=T, auc=T)

#Boot for SBP AUC/CCR

x<-roc.sbp[[i]]$original.predictor

y<-roc.sbp[[i]]$original.response

m<-data.frame(x, y)

boot<-boot(m, x="x", y="y", R=2000, statistic=fx.auc)

boot.auc.sbp[,i]<-boot$t

bootraw.auc.sbp[[i]]<-boot

boot<-boot(m, x="x", y="y", R=2000, statistic=fx.ccr)

boot.ccr.sbp[,i]<-boot$t

bootraw.ccr.sbp[[i]]<-boot

#Boot for DBP AUC/CCR

x<-roc.dbp[[i]]$original.predictor

y<-roc.dbp[[i]]$original.response

m<-data.frame(x, y)

boot<-boot(m, x="x", y="y", R=2000, statistic=fx.auc)

boot.auc.dbp[,i]<-boot$t

bootraw.auc.dbp[[i]]<-boot

boot<-boot(m, x="x", y="y", R=2000, statistic=fx.ccr)

boot.ccr.dbp[,i]<-boot$t

bootraw.ccr.dbp[[i]]<-boot

}

names(roc.sbp)<-sd_label

names(roc.dbp)<-sd_label

colnames(boot.auc.sbp)<-sd_label

colnames(boot.auc.dbp)<-sd_label

names(bootraw.auc.sbp)<-sd_label

names(bootraw.auc.dbp)<-sd_label

colnames(boot.ccr.sbp)<-sd_label

colnames(boot.ccr.dbp)<-sd_label

names(bootraw.ccr.sbp)<-sd_label

names(bootraw.ccr.dbp)<-sd_label

roc.sbp.all[[h]]<-roc.sbp

roc.dbp.all[[h]]<-roc.dbp

boot.auc.sbp.all[[h]]<-boot.auc.sbp

boot.auc.dbp.all[[h]]<-boot.auc.dbp

bootraw.auc.sbp.all[[h]]<-bootraw.auc.sbp

bootraw.auc.dbp.all[[h]]<-bootraw.auc.dbp

boot.ccr.sbp.all[[h]]<-boot.ccr.sbp

boot.ccr.dbp.all[[h]]<-boot.ccr.dbp

bootraw.ccr.sbp.all[[h]]<-bootraw.ccr.sbp

bootraw.ccr.dbp.all[[h]]<-bootraw.ccr.dbp

}

roc.sbp.k16<-roc.sbp.all[[1]]; roc.sbp.k20<-roc.sbp.all[[2]]; roc.sbp.k24<-roc.sbp.all[[3]]

roc.dbp.k16<-roc.dbp.all[[1]]; roc.dbp.k20<-roc.dbp.all[[2]]; roc.dbp.k24<-roc.dbp.all[[3]]

boot.auc.sbp.k16<-boot.auc.sbp.all[[1]]; bootraw.auc.sbp.k16<-bootraw.auc.sbp.all[[1]]

boot.auc.sbp.k20<-boot.auc.sbp.all[[2]]; bootraw.auc.sbp.k20<-bootraw.auc.sbp.all[[2]]

boot.auc.sbp.k24<-boot.auc.sbp.all[[3]]; bootraw.auc.sbp.k24<-bootraw.auc.sbp.all[[3]]

boot.auc.dbp.k16<-boot.auc.dbp.all[[1]]; bootraw.auc.dbp.k16<-bootraw.auc.dbp.all[[1]]

boot.auc.dbp.k20<-boot.auc.dbp.all[[2]]; bootraw.auc.dbp.k20<-bootraw.auc.dbp.all[[2]]

boot.auc.dbp.k24<-boot.auc.dbp.all[[3]]; bootraw.auc.dbp.k24<-bootraw.auc.dbp.all[[3]]

boot.ccr.sbp.k16<-boot.ccr.sbp.all[[1]]; bootraw.ccr.sbp.k16<-bootraw.ccr.sbp.all[[1]]

boot.ccr.sbp.k20<-boot.ccr.sbp.all[[2]]; bootraw.ccr.sbp.k20<-bootraw.ccr.sbp.all[[2]]

boot.ccr.sbp.k24<-boot.ccr.sbp.all[[3]]; bootraw.ccr.sbp.k24<-bootraw.ccr.sbp.all[[3]]

boot.ccr.dbp.k16<-boot.ccr.dbp.all[[1]]; bootraw.ccr.dbp.k16<-bootraw.ccr.dbp.all[[1]]

boot.ccr.dbp.k20<-boot.ccr.dbp.all[[2]]; bootraw.ccr.dbp.k20<-bootraw.ccr.dbp.all[[2]]

boot.ccr.dbp.k24<-boot.ccr.dbp.all[[3]]; bootraw.ccr.dbp.k24<-bootraw.ccr.dbp.all[[3]]

###########################################################################################

###ROC output

ret<-c("thres", "sen", "spe", "ppv", "npv", "accuracy")

#AUC

auc.sbp.all<-list(NA, NA, NA); names(auc.sbp.all)<-k_label

auc.dbp.all<-list(NA, NA, NA); names(auc.dbp.all)<-k_label

for (h in 1:3) {

dl<-roc.sbp.all[[h]]

auc.sbp<-rbind(c(dl[[1]]$ci[2], dl[[1]]$ci[1], dl[[1]]$ci[3]),

c(dl[[2]]$ci[2], dl[[2]]$ci[1], dl[[2]]$ci[3]),

c(dl[[3]]$ci[2], dl[[3]]$ci[1], dl[[3]]$ci[3]),

c(dl[[4]]$ci[2], dl[[4]]$ci[1], dl[[4]]$ci[3])

)

colnames(auc.sbp)<-c("OR", "UpperCI", "LowerCI")

rownames(auc.sbp)<-paste0("SD=", SD)

auc.sbp.all[[h]]<-auc.sbp

dl<-roc.dbp.all[[h]]

auc.dbp<-rbind(c(dl[[1]]$ci[2], dl[[1]]$ci[1], dl[[1]]$ci[3]),

c(dl[[2]]$ci[2], dl[[2]]$ci[1], dl[[2]]$ci[3]),

c(dl[[3]]$ci[2], dl[[3]]$ci[1], dl[[3]]$ci[3]),

c(dl[[4]]$ci[2], dl[[4]]$ci[1], dl[[4]]$ci[3])

)

colnames(auc.dbp)<-c("OR", "UpperCI", "LowerCI")

rownames(auc.dbp)<-paste0("SD=", SD)

auc.dbp.all[[h]]<-auc.dbp

}

print(auc.dbp.all)

print(auc.dbp.all)

#Threshold, Sens, Spec, PPV, NPV, accuracy

coordz.sbp.all<-list(NA, NA, NA); names(coordz.sbp.all)<-k_label

coordz.dbp.all<-list(NA, NA, NA); names(coordz.dbp.all)<-k_label

for (h in 1:3) {

dl<-roc.sbp.all[[h]]

coordz.sbp<-rbind(coords(dl[[1]], x=(K[h]-1)/2, ret=ret),

coords(dl[[2]], x=(K[h]-1)/2, ret=ret),

coords(dl[[3]], x=(K[h]-1)/2, ret=ret),

coords(dl[[4]], x=(K[h]-1)/2, ret=ret)

)

rownames(coordz.sbp)<-paste0("SD=", SD)

coordz.sbp.all[[h]]<-coordz.sbp

dl<-roc.dbp.all[[h]]

coordz.dbp<-rbind(coords(dl[[1]], x=(K[h]-1)/2, ret=ret),

coords(dl[[2]], x=(K[h]-1)/2, ret=ret),

coords(dl[[3]], x=(K[h]-1)/2, ret=ret),

coords(dl[[4]], x=(K[h]-1)/2, ret=ret)

)

rownames(coordz.dbp)<-paste0("SD=", SD)

coordz.dbp.all[[h]]<-coordz.dbp

}

print(coordz.sbp.all)

print(coordz.dbp.all)

#############################################################################################

####Figure 1 statistics

f.c_index<-formula(c_index~SD*k+(1|GID))

f.ccr<-formula(ccr~SD*k+(1|GID))

fx.mt<-function(x){

mt<-rbind(cbind(x[["k=16"]][,"SD=5"], rep(0, 2000), rep(0, 2000), rep( 1, 2000)),

cbind(x[["k=16"]][,"SD=10"], rep(1, 2000), rep(0, 2000), rep( 2, 2000)),

cbind(x[["k=16"]][,"SD=15"], rep(2, 2000), rep(0, 2000), rep( 3, 2000)),

cbind(x[["k=16"]][,"SD=20"], rep(3, 2000), rep(0, 2000), rep( 4, 2000)),

cbind(x[["k=20"]][,"SD=5"], rep(0, 2000), rep(1, 2000), rep( 5, 2000)),

cbind(x[["k=20"]][,"SD=10"], rep(1, 2000), rep(1, 2000), rep( 6, 2000)),

cbind(x[["k=20"]][,"SD=15"], rep(2, 2000), rep(1, 2000), rep( 7, 2000)),

cbind(x[["k=20"]][,"SD=20"], rep(3, 2000), rep(1, 2000), rep( 8, 2000)),

cbind(x[["k=24"]][,"SD=5"], rep(0, 2000), rep(2, 2000), rep( 9, 2000)),

cbind(x[["k=24"]][,"SD=10"], rep(1, 2000), rep(2, 2000), rep(10, 2000)),

cbind(x[["k=24"]][,"SD=15"], rep(2, 2000), rep(2, 2000), rep(11, 2000)),

cbind(x[["k=24"]][,"SD=20"], rep(3, 2000), rep(2, 2000), rep(12, 2000))

)

return(mt)

}

#C-index-SBP

mt<-fx.mt(boot.auc.sbp.all)

id<-seq(1, 12*2000, by=1)

mt<-cbind(id, mt)

colnames(mt)<-c("ID", "c_index", "SD", "k", "GID")

mt.auc.sbp<-as.data.frame(mt)

mt.auc.sbp$GID<-as.factor(mt.auc.sbp$GID)

lmer.auc.sbp<-lmer(formula = f.c_index, data=mt.auc.sbp)

summary(lmer.auc.sbp)

Anova(lmer.auc.sbp, type="III")

#C-index-DBP

mt<-fx.mt(boot.auc.dbp.all)

id<-seq(1, 12*2000, by=1)

mt<-cbind(id, mt)

colnames(mt)<-c("ID", "c_index", "SD", "k", "GID")

mt.auc.dbp<-as.data.frame(mt)

mt.auc.dbp$GID<-as.factor(mt.auc.dbp$GID)

lmer.auc.dbp<-lmer(formula = f, data=mt.auc.dbp)

summary(lmer.auc.dbp)

Anova(lmer.auc.dbp, type="III")

#CCR-SBP

mt<-fx.mt(boot.ccr.sbp.all)

id<-seq(1, 12*2000, by=1)

mt<-cbind(id, mt)

colnames(mt)<-c("ID", "ccr", "SD", "k", "GID")

mt.ccr.sbp<-as.data.frame(mt)

mt.ccr.sbp$GID<-as.factor(mt.ccr.sbp$GID)

lmer.ccr.sbp<-lmer(formula = f.ccr, data=mt.ccr.sbp)

summary(lmer.ccr.sbp)

Anova(lmer.ccr.sbp, type="III")

#CCR-DBP

mt<-fx.mt(boot.ccr.dbp.all)

id<-seq(1, 12*2000, by=1)

mt<-cbind(id, mt)

colnames(mt)<-c("ID", "ccr", "SD", "k", "GID")

mt.ccr.dbp<-as.data.frame(mt)

mt.ccr.dbp$GID<-as.factor(mt.ccr.dbp$GID)

lmer.ccr.dbp<-lmer(formula = f.ccr, data=mt.ccr.dbp)

summary(lmer.ccr.dbp)

Anova(lmer.ccr.dbp, type="III")

#############################################################################################

##Figure 1

col<-c(rgb(1,0,0,1), rgb(0,1,0,1), rgb(0,0,1,1))

x<-1:4

fx.plot<-function(x, y){

plot(x-0.16, y[["k=16"]][,"AUC"], ylim = c(0.7, 1), xlim=c(0.5, 4.5),

pch=16, col=col[1], cex=1.5, frame=F, xaxt="n", yaxt="n", type="b")

axis(side=1, at=c(1,2,3,4))

axis(side=2, at=c(0.7, 0.8, 0.9, 1.0))

segments(x0=x-0.16, x1=x-0.16,

y0=y[["k=16"]][,"Upper"], y1=y[["k=16"]][,"Lower"], col=col[1])

segments(x0=x-0.16+0.05, x1=x-0.16-0.05,

y0=y[["k=16"]][,"Upper"], y1=y[["k=16"]][,"Upper"], col=col[1])

segments(x0=x-0.16+0.05, x1=x-0.16-0.05,

y0=y[["k=16"]][,"Lower"], y1=y[["k=16"]][,"Lower"], col=col[1])

lines(x, y[["k=20"]][,"AUC"], cex=1.5, pch=16, col=col[2], type="b")

segments(x0=x, x1=x, y0=y[["k=20"]][,"Upper"],

y1=y[["k=20"]][,"Lower"], col=col[2])

segments(x0=x+0.05, x1=x-0.05,

y0=y[["k=20"]][,"Upper"], y1=y[["k=20"]][,"Upper"], col=col[2])

segments(x0=x+0.05, x1=x-0.05,

y0=y[["k=20"]][,"Lower"], y1=y[["k=20"]][,"Lower"], col=col[2])

lines(x+0.16, y[["k=24"]][,"AUC"], cex=1.5, pch=16, col=col[3], type="b")

segments(x0=x+0.16, x1=x+0.16,

y0=y[["k=24"]][,"Upper"], y1=y[["k=24"]][,"Lower"], col=col[3])

segments(x0=x+0.16+0.05, x1=x+0.16-0.05,

y0=y[["k=24"]][,"Upper"], y1=y[["k=24"]][,"Upper"], col=col[3])

segments(x0=x+0.16+0.05, x1=x+0.16-0.05,

y0=y[["k=24"]][,"Lower"], y1=y[["k=24"]][,"Lower"], col=col[3])

}

fx.list<-function(output){

y.list<-list(NA, NA, NA); names(y.list)<-k_label

for (h in 1:3) {

auc<-c(median(unlist(output[[h]][["SD=5"]]["t"])),

median(unlist(output[[h]][["SD=10"]]["t"])),

median(unlist(output[[h]][["SD=15"]]["t"])),

median(unlist(output[[h]][["SD=20"]]["t"])))

upper<-rep(NA, 4)

lower<-rep(NA, 4)

for(i in 1:4) {

boot.ci<-boot.ci(output[[h]][[i]], type="perc")

lower[i]<-boot.ci$percent[4]

upper[i]<-boot.ci$percent[5]

}

y.list[[h]]<-matrix(c(auc, lower, upper),

ncol = 3, nrow = 4,

dimnames = list(sd_label, c("AUC", "Lower", "Upper"))

)

}

return(y.list)

}

##SBP.AUC

y.sbp.auc<-fx.list(bootraw.auc.sbp.all)

print(y.sbp.auc)

fx.plot (x, y.sbp.auc)

##DBP.AUC

y.dbp.auc<-fx.list(bootraw.auc.dbp.all)

print(y.dbp.auc)

fx.plot (x, y.dbp.auc)

##SBP.CCR

y.sbp.ccr<-fx.list(bootraw.ccr.sbp.all)

print(y.sbp.ccr)

fx.plot (x, y.sbp.ccr)

##DBP.CCR

y.dbp.ccr<-fx.list(bootraw.ccr.dbp.all)

print(y.dbp.ccr)

fx.plot (x, y.dbp.ccr)

#######################################################################################

#Figure 2

fx.fit<-function(list, k, sd, x, y){

x<-as.numeric(list[[k]][[x]][,sd])

y<-as.numeric(list[[k]][[y]][,sd])

plot(x, y, pch=16, ylim=c(120, 150), xlim=c(0, 24), xaxt="n", yaxt="n", frame=F)

abline(h=135, v=12, col="blue", lty=2)

axis(side=1, seq(0, 24, by =4))

axis(side=2, seq(120, 150, by=5))

lm<-lm(y~x)

abline(lm$coef, col="blue")

P<-predict(lm, interval="prediction")

polygon(x=c(x[order(x)], rev(x[order(x)])),

y=c(P[,2][order(x)], rev(P[,3][order(x)])),

col=rgb(1, 0, 0, 0.1), border = F)

xP<-cbind(x, P)

up.dw<-c(min(xP[order(x),"x"][which(xP[order(x),"lwr"]>=135)]),

max(xP[order(x),"x"][which(xP[order(x),"upr"]<135)]))

abline(v=up.dw, lty=2, col="gray")

out<-list(lm, xP, up.dw); names(out)<-c("lm","xP", "up.dw")

return(out)

}

###SBP

#SD=5

k24.sd5.sbp<-fx.fit(bp.list.all, k="k=24", sd="SD=5", x="nsbp135", y="meansbp")

summary(k24.sd5.sbp$lm)

k24.sd5.sbp$xP

k24.sd5.sbp$up.dw

#SD=10

k24.sd10.sbp<-fx.fit(bp.list.all, k="k=24", sd="SD=10", x="nsbp135", y="meansbp")

summary(k24.sd10.sbp$lm)

k24.sd10.sbp$xP

k24.sd10.sbp$up.dw

#SD=15

k24.sd15.sbp<-fx.fit(bp.list.all, k="k=24", sd="SD=15", x="nsbp135", y="meansbp")

summary(k24.sd15.sbp$lm)

k24.sd15.sbp$xP

k24.sd15.sbp$up.dw

#SD=20

k24.sd20.sbp<-fx.fit(bp.list.all, k="k=24", sd="SD=20", x="nsbp135", y="meansbp")

summary(k24.sd20.sbp$lm)

k24.sd20.sbp$xP

k24.sd20.sbp$up.dw

###DBP

#SD=5

k24.sd5.dbp<-fx.fit(bp.list.all, k="k=24", sd="SD=5", x="nsbp135", y="meansbp")

summary(k24.sd5.dbp$lm)

k24.sd5.dbp$xP

k24.sd5.dbp$up.dw

#SD=10

k24.sd10.dbp<-fx.fit(bp.list.all, k="k=24", sd="SD=10", x="nsbp135", y="meansbp")

summary(k24.sd10.dbp$lm)

k24.sd10.dbp$xP

k24.sd10.dbp$up.dw

#SD=15

k24.sd15.dbp<-fx.fit(bp.list.all, k="k=24", sd="SD=15", x="nsbp135", y="meansbp")

summary(k24.sd15.dbp$lm)

k24.sd15.dbp$xP

k24.sd15.dbp$up.dw

#SD=20

k24.sd20.dbp<-fx.fit(bp.list.all, k="k=24", sd="SD=20", x="nsbp135", y="meansbp")

summary(k24.sd20.dbp$lm)

k24.sd20.dbp$xP

k24.sd20.dbp$up.dw

*For the validation cohort*

m<-read.csv("HBPM_424_pt.csv", header = T)

m<-subset(m, nmeasure>=8)

####Code book

# "id" : ID

# "sex" : Sex (Female =0, male =1)

# "age" : Age (years)

# "bmi" : Body mass index (kg/m2)

# "dm" : Diabetes mellitus (yes=1, no=0)

# "dyslipidemia": Dyslipidemia (yes=1, no=0)

# "nmeasure": number of BP readings

# "mean.sbp": mean SBP (mmHg)

# "mean.dbp" : mean DBP (mmHg)

# "sd.sbp": SD of SBP (mmHg)

# "sd.dbp": SD of DBP (mmHg)

# "n.sbp135": number of SBP>135 mmHg (high SBP count)

# "n.dbp85": number of DBP>85 mmHg (high DBP count)

# "n.htn.135_85": number of BP >135/85 mmHg (high BP count)

# "maxsbp": maximum SBP among SBP readings

# "maxdbp": maximum DBP among DBP readings

# "minsbp": minimum SBP among SBP readings

# "mindbp": minimum DBP among DBP readings

# "htn": hypertension (yes=1, no=0)

# "sys.htn": systolic hypertension (mean SBP>=135 mmHg; yes=1, no=0)

# "dia.htn": diastolic hypertension (yes=1, no=0)

#Data structure

hist(m$nmeasure, 20)

summary(m$mean.sbp); sd(m$mean.sbp)

summary(m$mean.dbp); sd(m$mean.dbp)

table(m$sys.htn)

m$rate.measure.135_85<-m$n.htn.135_85/m$nmeasure

hist(m$rate.measure.135_85, 20)

summary(m$rate.measure.135_85); sd(m$rate.measure.135_85)

######################################################################################

summary(m$age); sd(m$age, na.rm = T)

table(m$sex); table(m$sex)/NROW(m)

summary(m$bmi); sd(m$bmi, na.rm = T)

table(m$dm); table(m$dm)/NROW(m)

table(m$dyslipidemia); table(m$dyslipidemia)/NROW(m)

table(m$htn); table(m$htn)/NROW(m)

table(m$sys.htn); table(m$sys.htn)/NROW(m)

table(m$dia.htn); table(m$dia.htn)/NROW(m)

m$sbp130_140<-ifelse(m$mean.sbp<140&m$mean.sbp>=130,1, 0)

table(m$sbp130_140); table(m$sbp130_140)/NROW(m)

m$dbp80_90<-ifelse(m$mean.dbp<90&m$mean.dbp>=80,1, 0)

table(m$dbp80_90); table(m$dbp80_90)/NROW(m)

#####################################################################################

ret<-c("threshold", "sens", "spec", "ppv", "npv", "accuracy")

#ROC curve with ratio of SBP>=135

table(m$sys.htn); table(m$sys.htn)/NROW(m)

(m$rate.sbp135<-m$n.sbp135/m$nmeasure)

hist(m$rate.sbp135, 20)

roc.sbp.rate<-roc(sys.htn~rate.sbp135, data=m, ci=T, auc=T)

roc.sbp.rate

plot(1-roc.sbp.rate$specificities, roc.sbp.rate$sensitivities, type = "l")

abline(c(0,1), c(1,1), lty=2)

coordx<-coords(roc.sbp.rate, ret=ret)

Jx<-coordx$sensitivity+coordx$specificity

coordx[which(Jx==max(Jx)),]

coordx[coordx$threshold==max(coordx$threshold[which(coordx$threshold<=0.50)]),]

#ROC curve with ratio of dbp>85

table(m$dia.htn); table(m$dia.htn)/NROW(m)

m$rate.dbp85<-m$n.dbp85/m$nmeasure

hist(m$rate.dbp85, 20)

roc.dbp.rate<-roc(dia.htn~rate.dbp85, data=m, ci=T, auc=T)

roc.dbp.rate

plot(1-roc.dbp.rate$specificities, roc.dbp.rate$sensitivities, type = "l")

abline(c(0,1), c(1,1), lty=2)

coordx<-coords(roc.dbp.rate, ret=ret)

Jx<-coordx$sensitivity+coordx$specificity

coordx[which(Jx==max(Jx)),]

coordx[coordx$threshold==max(coordx$threshold[which(coordx$threshold<=0.50)]),]

##########################################################################################

####Overall high BP count/number of BP reading (count/total ratio; C/T ratio)

#For SBP

m$n.sbp135.bi<-ifelse(m$rate.sbp135>=0.5, 1, 0)

roc.n.sbp<-roc(sys.htn~n.sbp135.bi, data=m, ci=T, auc=T)

roc.n.sbp

coordx.n.sbp<-coords(roc.n.sbp, ret=ret)

coordx.n.sbp[2,]

#For dBP

m$n.dbp85.bi<-ifelse(m$rate.dbp85>=0.5, 1, 0)

roc.n.dbp<-roc(dia.htn~n.dbp85.bi, data=m, ci=T, auc=T)

roc.n.dbp

coordx.n.dbp<-coords(roc.n.dbp, ret=ret)

coordx.n.dbp[2,]

#########################################################################################

###according to the number of measurements

m$nmeasure3g<-1

m$nmeasure3g[m$nmeasure<16]<-0

m$nmeasure3g[m$nmeasure>=24]<-2

table(m$nmeasure3g)

#nmeasure3g==0

roc.sys.rate.g0<-roc(sys.htn~rate.sbp135, data=m[m$nmeasure3g==0,], ci=T, auc=T)

roc.sys.rate.g0

with(m[m$nmeasure3g==0,], CrossTable(sys.htn, n.sbp135.bi, chisq = T))

roc.g0.sbp<-roc(sys.htn~n.sbp135.bi, data=m[m$nmeasure3g==0,], ci=T, auc=T)

roc.g0.sbp

coordx.g0.sbp<-coords(roc.g0.sbp, ret=ret)

coordx.g0.sbp

#nmeasure3g==1

roc.sys.rate.g1<-roc(sys.htn~rate.sbp135, data=m[m$nmeasure3g==1,], ci=T, auc=T)

roc.sys.rate.g1

with(m[m$nmeasure3g==1,], CrossTable(sys.htn, n.sbp135.bi, chisq = T))

roc.g1.sbp<-roc(sys.htn~n.sbp135.bi, data=m[m$nmeasure3g==1,], ci=T, auc=T)

roc.g1.sbp

coordx.g1.sbp<-coords(roc.g1.sbp, ret=ret)

coordx.g1.sbp

#nmeasure3g==2

roc.sys.rate.g2<-roc(sys.htn~rate.sbp135, data=m[m$nmeasure3g==2,], ci=T, auc=T)

roc.sys.rate.g2

with(m[m$nmeasure3g==2,], CrossTable(sys.htn, n.sbp135.bi, chisq = T))

roc.g2.sbp<-roc(sys.htn~n.sbp135.bi, data=m[m$nmeasure3g==2,], ci=T, auc=T)

roc.g2.sbp

coordx.g2.sbp<-coords(roc.g2.sbp, ret=ret)

coordx.g2.sbp

#####################################################################################

###according to the standard deviations of SBP

hist(m$sd.sbp, 20)

table(m$sd.sbp<=5)

m$sd.sbp.3g<-1

m$sd.sbp.3g[m$sd.sbp<10]<-0

m$sd.sbp.3g[m$sd.sbp>=15]<-2

table(m$sd.sbp.3g)

#SD=10

with(m[m$sd.sbp.3g==0,], CrossTable(sys.htn, n.sbp135.bi, chisq = T))

roc.sys.rate.sg0<-roc(sys.htn~rate.sbp135, data=m[m$sd.sbp.3g==0,], ci=T, auc=T)

roc.sys.rate.sg0

roc.sg0.sbp<-roc(sys.htn~n.sbp135.bi, data=m[m$sd.sbp.3g==0,], ci=T, auc=T)

roc.sg0.sbp

coordx.sg0.sbp<-coords(roc.sg0.sbp, ret=ret)

coordx.sg0.sbp

#SD=10-15

with(m[m$sd.sbp.3g==1,], CrossTable(sys.htn, n.sbp135.bi, chisq = T))

roc.sys.rate.sg1<-roc(sys.htn~rate.sbp135, data=m[m$sd.sbp.3g==1,], ci=T, auc=T)

roc.sys.rate.sg1

roc.sg1.sbp<-roc(sys.htn~n.sbp135.bi, data=m[m$sd.sbp.3g==1,], ci=T, auc=T)

roc.sg1.sbp

coordx.sg1.sbp<-coords(roc.sg1.sbp, ret=ret)

coordx.sg1.sbp

#SD>=15

with(m[m$sd.sbp.3g==2,], CrossTable(sys.htn, n.sbp135.bi, chisq = T))

roc.sys.rate.sg2<-roc(sys.htn~rate.sbp135, data=m[m$sd.sbp.3g==2,], ci=T, auc=T)

roc.sys.rate.sg2

roc.sg2.sbp<-roc(sys.htn~n.sbp135.bi, data=m[m$sd.sbp.3g==2,], ci=T, auc=T)

roc.sg2.sbp

coordx.sg2.sbp<-coords(roc.sg2.sbp, ret=ret)

coordx.sg2.sbp

##########################################################################################

###according to the max range of SBP

hist(m$maxsbp-m$minsbp)

m$range.sbp<-m$maxsbp-m$minsbp

m$range.sbp.3g<-1

m$range.sbp.3g[m$range.sbp<40]<-0

m$range.sbp.3g[m$range.sbp>=60]<-2

table(m$range.sbp.3g)

#Max diff <40mmHg

with(m[m$range.sbp.3g==0,], CrossTable(sys.htn, n.sbp135.bi, chisq = T))

roc.sys.rate.rg0<-roc(sys.htn~rate.sbp135, data=m[m$range.sbp.3g==0,], ci=T, auc=T)

roc.sys.rate.rg0

roc.rg0.sbp<-roc(sys.htn~n.sbp135.bi, data=m[m$range.sbp.3g==0,], ci=T, auc=T)

roc.rg0.sbp

coordx.rg0.sbp<-coords(roc.rg0.sbp, ret=ret)

coordx.rg0.sbp

#Max diff 40-60mmHg

with(m[m$range.sbp.3g==1,], CrossTable(sys.htn, n.sbp135.bi, chisq = T))

roc.sys.rate.rg1<-roc(sys.htn~rate.sbp135, data=m[m$range.sbp.3g==1,], ci=T, auc=T)

roc.sys.rate.rg1

roc.rg1.sbp<-roc(sys.htn~n.sbp135.bi, data=m[m$range.sbp.3g==1,], ci=T, auc=T)

roc.rg1.sbp

coordx.rg1.sbp<-coords(roc.rg1.sbp, ret=ret)

coordx.rg1.sbp

#max diff >60mmHg

with(m[m$range.sbp.3g==2,], CrossTable(sys.htn, n.sbp135.bi, chisq = T))

roc.sys.rate.rg2<-roc(sys.htn~rate.sbp135, data=m[m$range.sbp.3g==2,], ci=T, auc=T)

roc.sys.rate.rg2

roc.rg2.sbp<-roc(sys.htn~n.sbp135.bi, data=m[m$range.sbp.3g==2,], ci=T, auc=T)

roc.rg2.sbp

coordx.rg2.sbp<-coords(roc.rg2.sbp, ret=ret)

coordx.rg2.sbp

#############################################################################################

###according to the mean SBP

hist(m$mean.sbp)

m$mean.sbp.3g<-1

m$mean.sbp.3g[m$mean.sbp<130]<-0

m$mean.sbp.3g[m$mean.sbp>=140]<-2

table(m$mean.sbp.3g)

#mean BP

with(m[m$mean.sbp.3g==0,], CrossTable(sys.htn, n.sbp135.bi, chisq = T))

with(m[m$mean.sbp.3g==1,], CrossTable(sys.htn, n.sbp135.bi, chisq = T))

roc.sys.rate.mg1<-roc(sys.htn~rate.sbp135, data=m[m$mean.sbp.3g==1,], ci=T, auc=T)

roc.sys.rate.mg1

roc.mg1.sbp<-roc(sys.htn~n.sbp135.bi, data=m[m$mean.sbp.3g==1,], ci=T, auc=T)

roc.mg1.sbp

coordx.mg1.sbp<-coords(roc.mg1.sbp, ret=ret)

coordx.mg1.sbp

with(m[m$mean.sbp.3g==2,], CrossTable(sys.htn, n.sbp135.bi, chisq = T))

###########################################################################################

### count-to-total ratio; C/T ratio

m$rate.sbp135<-m$n.sbp135/m$nmeasure

m$rate.dbp85<-m$n.dbp85/m$nmeasure

##SBP

plot(m$rate.sbp135, m$mean.sbp, frame=F, pch=16)

abline(v=0.5, h=135, lty=2, col="blue")

lm.sbp.log<-lm(mean.sbp~log(rate.sbp135/(1-rate.sbp135)),

data=m[m$rate.sbp135>0&m$rate.sbp135<1,])

summary(lm.sbp.log)

P.sbp.log<-predict(lm.sbp.log, interval="predict")

P.sbp.log<-as.data.frame(P.sbp.log)

rate<-m$rate.sbp135[m$rate.sbp135>0&m$rate.sbp135<1]

P.sbp.log<-cbind(P.sbp.log, rate)

#Graph

x<-seq(0.001, 0.999, by=0.001)

est<-lm.sbp.log$coefficients

y<-est[2]*log(x/(1-x))+est[1]

lines(x, y, type = "l", col="red")

x<-m$rate.sbp135[m$rate.sbp135>0&m$rate.sbp135<1]

polygon(x=c(x[order(x)], rev(x[order(x)])),

y=c(P.sbp.log$lwr[order(x)], rev(P.sbp.log$upr[order(x)])),

border = F, col=rgb(1,0,0, 0.1))

cbind(x[order(x)], P.sbp.log[order(x),])

abline(v=x[order(x)][max(which(P.sbp.log$upr[order(x)]<135))], lty=2, col="black")

abline(v=x[order(x)][min(which(P.sbp.log$lwr[order(x)]>=135))], lty=2, col="black")

P.sbp.log$width<-P.sbp.log$upr-P.sbp.log$lwr

mean(P.sbp.log$width)

#SBP between C/T ratio of 0.2 and C/T ratio of 0.8

point.sbp.rate<-rbind(P.sbp.log[which(P.sbp.log$rate==0.2),][1,],

P.sbp.log[which(P.sbp.log$rate==0.3),][1,],

P.sbp.log[which(P.sbp.log$rate==0.4),][1,],

P.sbp.log[which(P.sbp.log$rate==0.5),][1,],

P.sbp.log[which(P.sbp.log$rate==0.6),][1,],

P.sbp.log[which(P.sbp.log$rate==0.7),][1,],

P.sbp.log[which(P.sbp.log$rate==0.8),][1,])

rownames(point.sbp.rate)<-as.character(seq(0.2, 0.8, by=0.1))

point.sbp.rate

##DBP

plot(m$rate.dbp85, m$mean.dbp, frame=F, pch=16)

abline(v=0.5, h=85, lty=2, col="blue")

lm.dbp.log<-lm(mean.dbp~log(rate.dbp85/(1-rate.dbp85)), data=m[m$rate.dbp85>0&m$rate.dbp85<1,])

summary(lm.dbp.log)

P.dbp.log<-predict(lm.dbp.log, interval="predict")

P.dbp.log<-as.data.frame(P.dbp.log)

rate<-m$rate.dbp85[m$rate.dbp85>0&m$rate.dbp85<1]

P.dbp.log<-cbind(P.dbp.log, rate)

#Graph

x<-seq(0.001, 0.999, by=0.001)

est<-lm.dbp.log$coefficients

y<-est[2]*log(x/(1-x))+est[1]

lines(x, y, type = "l", col="red")

x<-m$rate.dbp85[m$rate.dbp85>0&m$rate.dbp85<1]

polygon(x=c(x[order(x)], rev(x[order(x)])),

y=c(P.dbp.log$lwr[order(x)], rev(P.dbp.log$upr[order(x)])),

border = F, col=rgb(1,0,0, 0.1))

cbind(x[order(x)], P.dbp.log[order(x),])

low<-x[order(x)][max(which(P.dbp.log$upr[order(x)]<85))]

abline(v=low, lty=2, col="black")

high<-x[order(x)][min(which(P.dbp.log$lwr[order(x)]>=85))]

abline(v=high, lty=2, col="black")

P.dbp.log$width<-P.dbp.log$upr-P.dbp.log$lwr

mean(P.dbp.log$width)

#DBP between C/T ratio of 0.2 and C/T ratio of 0.8

point.dbp.rate<-rbind(P.dbp.log[which(P.dbp.log$rate==0.2),][1,],

P.dbp.log[which(P.dbp.log$rate==0.3),][1,],

P.dbp.log[which(P.dbp.log$rate==0.4),][1,],

P.dbp.log[which(P.dbp.log$rate==0.5),][1,],

P.dbp.log[which(P.dbp.log$rate==0.6),][1,],

P.dbp.log[which(P.dbp.log$rate==0.7),][1,],

P.dbp.log[which(P.dbp.log$rate==0.8),][1,])

rownames(point.dbp.rate)<-as.character(seq(0.2, 0.8, by=0.1))

point.dbp.rate

############################################################################################

###relationship between C/T ratio and mean BPs in the restricted range

##SBP

(r.sbp<-which(m$mean.sbp >125&m$mean.sbp < 145))

mean(m$sd.sbp[r.sbp])

lm.rsbp<-lm(mean.sbp~rate.sbp135, x=T, y=T, data=m[r.sbp,])

summary(lm.rsbp)

p.rsbp<-predict(lm.rsbp, interval = "predict", se.fit = T)

rate.seq<-data.frame(rate.sbp135=seq(0.2, 0.8, by=0.1))

sbp.seq<-predict(lm.rsbp, newdata=rate.seq, interval = "predict")

rownames(sbp.seq)<-as.character(unlist(rate.seq))

with (m[r.sbp,], plot(rate.sbp135, mean.sbp, frame=F,

pch=16, ylim = c(123, 147),

xlim=c(0.1, 0.9), xaxt="n", yaxt="n")

)

axis(side=1, at=seq(0.2, 0.8, by=0.2))

axis(side=2, at=seq(125, 145, by=5))

abline(lm.rsbp$coefficients, col="red")

x<-lm.rsbp$x[,2]

yu<-p.rsbp$fit[,"upr"][order(x)]

yl<-p.rsbp$fit[,"lwr"][order(x)]

mean(yu-yl)

polygon(x=c(sort(x), sort(x, decreasing = T)), y=c(yu, rev(yl)),

col=rgb(1, 0, 0, 0.1), border = F)

abline(h=135, v=0.5, lty=2, col="blue")

(low.rate<-max(sort(x)[which(as.vector(yu)<135)]))

(high.rate<-min(sort(x)[which(as.vector(yl)>135)]))

abline(v=c(low.rate, high.rate), lty=2, col="gray")

##DBP

(r.dbp<-which(m$mean.dbp >75&m$mean.dbp < 95))

mean(m$sd.dbp[r.dbp])

lm.rdbp<-lm(mean.dbp~rate.dbp85, x=T, y=T, data=m[r.dbp,])

summary(lm.rdbp)

p.rdbp<-predict(lm.rdbp, interval = "predict", se.fit = T)

rate.seq<-data.frame(rate.dbp85=seq(0.2, 0.8, by=0.1))

dbp.seq<-predict(lm.rdbp, newdata=rate.seq, interval = "predict")

rownames(dbp.seq)<-as.character(unlist(rate.seq))

with (m[r.dbp,], plot(rate.dbp85, mean.dbp, frame=F,

pch=16, ylim = c(73, 97), xlim=c(0.1, 0.9), xaxt="n", yaxt="n")

)

axis(side=1, at=seq(0.2, 0.8, by=0.2))

axis(side=2, at=seq(75, 95, by=5))

abline(lm.rdbp$coefficients, col="red")

x<-lm.rdbp$x[,2]

yu<-p.rdbp$fit[,"upr"][order(x)]

yl<-p.rdbp$fit[,"lwr"][order(x)]

polygon(x=c(sort(x), sort(x, decreasing = T)), y=c(yu, rev(yl)),

col=rgb(1, 0, 0, 0.1), border = F)

mean(yu-yl)

abline(h=85, v=0.5, lty=2, col="blue")

(low.rate<-max(sort(x)[which(as.vector(yu)<85)]))

(high.rate<-min(sort(x)[which(as.vector(yl)>85)]))

abline(v=c(low.rate, high.rate), lty=2, col="gray")
